# Structure of Hypomanic Symptoms in Adolescents With Bipolar Disorders: A Network Approach

**DOI:** 10.3389/fpsyt.2022.844699

**Published:** 2022-04-18

**Authors:** Yuan Yang, Wu-Yang Zhang, Yao Zhang, Shuying Li, Teris Cheung, Dexing Zhang, Todd Jackson, Fan He, Yu-Tao Xiang

**Affiliations:** ^1^Guangdong Mental Health Center, Guangdong Academy of Medical Sciences, Guangdong Provincial People’s Hospital, Guangzhou, China; ^2^Department of Pediatric Development and Behavior, The Third Affiliated Hospital of Zhengzhou University, Zhengzhou, China; ^3^Huashan Hospital, Fudan University, Shanghai, China; ^4^Department of Psychiatry, The First Affiliated Hospital of Zhengzhou University, Zhengzhou, China; ^5^School of Nursing, Hong Kong Polytechnic University, Kowloon, Hong Kong SAR, China; ^6^Jockey Club School of Public Health and Primary Care, Faculty of Medicine, Chinese University of Hong Kong, Sha Tin, Hong Kong SAR, China; ^7^Department of Psychology, University of Macau, Taipa, Macao SAR, China; ^9^Unit of Psychiatry, Department of Public Health and Medicinal Administration, Institute of Translational Medicine, Faculty of Health Sciences, University of Macau, Macao, Macao SAR, China; ^8^The National Clinical Research Center for Mental Disorders & Beijing Key Laboratory of Mental Disorders, Beijing Anding Hospital and Advanced Innovation Center for Human Brain Protection, School of Mental Health, Capital Medical University, Beijing, China; ^10^Center for Cognition and Brain Sciences, University of Macau, Taipa, Macao SAR, China; ^11^Institute of Advanced Studies in Humanities and Social Sciences, University of Macau, Taipa, Macao SAR, China

**Keywords:** HCL-33, HCL-33-EA, network, adolescents, Chinese, bipolar, hypomanic

## Abstract

**Background:**

Bipolar disorders (BD) are severe mental illnesses that are often misdiagnosed or under-diagnosed. The self-report 33-item Hypomania Checklist (HCL-33) and the 33-item Hypomania Checklist – external assessment (HCL-33-EA) are well-validated scales for BD symptom detection. This study compared the network structure, central symptoms, and network stability of hypomanic symptoms measured by the HCL-33 vs. the HCL-33-EA.

**Methods:**

This cross-sectional study was conducted from January to December 2019. Adolescents (aged between 12 and 18 years) with BD were recruited from the outpatient department of Child Psychiatry, First Affiliated Hospital of Zhengzhou University. All participants were asked to complete the HCL-33, and their caregivers completed the HCL-33-EA. Network analyses were conducted.

**Results:**

A total of 215 adolescents with BD and their family caregivers were recruited. Node HCL17 (“talk more,” node strength = 4.044) was the most central symptom in the HCL-33 network, followed by node HCL2 (“more energetic,” node strength = 3.822), and HCL18 (“think faster,” node strength = 3.801). For the HCL-33-EA network model, node HCL27 (“more optimistic,” node strength = 3.867) was the most central node, followed by node HCL18 (“think faster,” node strength = 3.077), and HCL17 (“talk more,” node strength = 2.998). In the network comparison test, there was no significant difference at the levels of network structure (*M* = 0.946, *P* = 0.931), global strength (S: 5.174, *P* = 0.274), or each specific edge (all *P*’s > 0.05 after Holm–Bonferroni corrections) between HCL-33 and HCL-33-EA items. Network stabilities for both models were acceptable.

**Conclusion:**

The nodes “talk more” and “think faster” acted as central symptoms in BD symptom network models based on the HCL-33 and HCL-33-EA. Although the most prominent central symptom differed between the two models (“talk more” in HCL-33 vs. “more optimistic” in HCL-33-EA model), networks based on each measure were highly similar and underscored similarities in BD symptom relations perceived by adolescents and their caregivers. This research provides foundations for future studies with larger sample sizes toward improving the accuracy and robustness of observed network structures.

## Introduction

Bipolar disorders (BD) are a category of major mental illnesses that are often misdiagnosed as major depressive disorder (MDD) or under-diagnosed in clinical practice (1–3). According to a recent meta-analysis, there are an estimated 1.54 million people with BD in China (4). A previous study revealed that about 21% of BD patients in China report having been misdiagnosed in clinical practice (5). Consequently, it can take up to 10 years before the appropriate diagnosis is made, with consequences that include lowered treatment efficacy, and increased suicide risk (6, 7).

To reduce the likelihood of BD misdiagnosis, several diagnostic instruments have been developed including the clinician-rated Mini-International Neuropsychiatric Interview (M.I.N.I) (8), and the lay interviewer-rated Composite International Diagnostic Interview (CIDI) (9). However, these diagnostic tools are both time-consuming and expensive. Therefore, a number of brief, cost-saving self-report scales that assess clinical features of BD have also been developed, including the Hypomania Checklist (HCL) (10). The HCL is specifically designed to detect subtle BD symptoms in the domains of emotion, thinking, and behavior typically observed in hypomanic states (10). The HCL has been well-validated in various countries, with good psychometric properties (11).

The 33-item Hypomania Checklist (HCL-33) is a patient-rated screening instrument for hypomanic symptoms in past and/or current episodes and has been validated in various populations including Chinese adolescents and older adults (12, 13). Conversely, the 33-item Hypomania Checklist – external assessment (HCL-33-EA) is an observer-rated version of the HCL-33 that was designed to assess patients’ hypomanic symptoms based on ratings of their caregivers (14). The HCL-33 and HCL-33-EA are significantly and positively correlated with one another (15), though the HCL-33-EA is more sensitive in correctly distinguishing BD patients from MDD patients compared to the HCL-33 (16). To date, no study has examined the network structure of the HCL-33 or the HCL-33-EA. Previous studies typically focused singularly on HCL-33 total or mean scores without any attention to the relative importance and interrelations of specific symptoms. Consequently, investigating HCL scales at a symptom level using network analysis might provide new insights into the importance of different individual symptoms in relation to BD as a whole (17). Network analysis is a novel approach to examining the structure of psychopathology. Recently, several network analyses have been conducted on different psychiatric disorder categories including depression, anxiety, obsessive compulsive disorder, and eating disorders (18–20). For example, one study found that “self-hatred,” “loneliness,” “sadness,” and “pessimism” were the most central (influential) depressive symptoms in adolescents (17), while another study found death wishes were a key symptom that sustains depression (21). It has also been found that patients who endorse more central symptoms of depression at baseline have a greater chance of experiencing MDD in their later life compared to those who endorse more peripheral symptoms of depression at baseline (22).

In network analysis, higher centrality indicates greater importance (23). Analyzing the structure of symptoms measured by HCL scales from the perspective of network analysis would enable us to understand which symptoms might be particularly important in triggering and maintaining a broader range of hypomanic symptoms. The identification of central symptoms would also be potentially useful from the perspective of developing targeted interventions that address critical hypomania symptoms.

Hence, this study examined the structure of BD symptoms measured by the HCL-33, and the HCL-33-EA using a network approach. In addition, we compared the network structure, central symptoms, and network stability of network models generated on the basis of each HCL version.

## Materials and Methods

### Study Participants

This cross-sectional study was conducted from January to December 2019. All participants were consecutively recruited from the outpatient department of Child Psychiatry of a tertiary hospital, the First Affiliated Hospital of Zhengzhou University. To be eligible, all participants were: (1) aged between 12 and 18 years; (2) diagnosed with a BD according to the 10th Revision of the International Statistical Classification of Diseases and Related Health Problems (ICD-10) (24); and (3) able to understand Chinese and the contents of the assessments. Patients with acute manic episodes and those with severe medical or neurological conditions were excluded. Participants’ diagnosis, clinical status, and eligibility were confirmed by their treating psychiatrist. Additionally, participants’ caregivers (e.g., mother, father, sibling, or close friends) were invited to complete the HCL-33-EA. All participants provided verbal informed consent while their legal guardians provided written informed consent. The study protocol was approved by the Medical Ethics Committee of the First Affiliated Hospital of Zhengzhou University.

### Measurements

Participants’ and caregivers’ basic demographic data were collected. Chinese versions of the validated self-report HCL-33 (12, 13) and caregiver-rated HCL-33-EA (14, 15) were administered to assess the patient’s hypomanic symptoms. Both the HCL-33 and the HCL-33-EA consist of 33 symptom items with dichotomous response options to assess presence of a symptom (Yes/No). Total scores on these scales range from 0 to 33, with higher scores indicating more severe hypomanic symptoms. A previous comparative study found that the HCL-33-EA was more sensitive than the HCL-33 in distinguishing BD patients from MDD patients (0.83 vs. 0.59) while the HCL-33 presented better specificity than the HCL-33-EA did (0.82 vs. 0.68) (16).

### Network Estimation

All network analyses were conducted using R program (25). To estimate the network structure of hypomanic symptoms measured by the HCL-33 and HCL-33-EA, an Ising model was applied since all scale items (nodes) were dichotomous (26). In network analysis, each symptom is defined as a “*node*,” and the pairwise association between symptoms is defined as an “*edge*.” Nodes that are stronger or more connected with other nodes are located in the central area of the model. A thicker edge indicates a stronger correlation. Green edges indicate positive correlations while red edges indicate negative correlations (23). Following previous studies (18, 27), the “*estimateNetwork*” function was adopted to establish the network model, with 0.5 as the default tuning parameter (28).

### Network Centrality

As recommended previously (17, 29), in the subsequent network analysis we focused on the centrality index of strength (17) which is the total sum of absolute weights of the edge connecting a node to all other nodes (30). In addition, predictability, which qualifies how well a specific node is predicted by all its neighboring nodes, was estimated using R-package “*mgm*” (Version 1.2-11) (31).

### Network Stability and Accuracy

The stability and accuracy of each network model were assessed using R-package “*bootnet*” (28). First, a case-dropping bootstrap procedure was performed to compute correlation stability coefficients (CS-C) (1,000 replications). A CS-C is required to be above 0.25, and preferably 0.50 (28). Second, non-parametric bootstrapping was used to estimate the accuracy of edge-weights by computing confidence intervals (CIs). Larger CIs indicated poorer precision in the estimation of edges while narrower CIs indicated a more precise edge-weight network (17). Finally, differences in network properties (i.e., edge weights and node strengths) were evaluated via bootstrapped difference tests (28).

### Network Comparison

To compare the network characteristics of hypomanic symptom communities measured by the HCL-33 and the HCL-33-EA, respectively, we used the “*NetworkComparisonTest*” package. These analyses investigated possible differences between the two BD measures at the levels of network structure (i.e., edge weight distributions), global strength (i.e., overall absolute connectivity among the symptoms), and each specific edge (28). The package is a permutation-based test that randomly regroups participants from each network repeatedly (1,000 replications) and then examines the differences between networks (32). The general network structure invariance test explores differences in the network structure as a whole. In instances of significant differences observed between the two network structures, we tested for specific edges that displayed significant differences.

## Results

### Participant Characteristics

In total, 215 patients with BD (46 males and 169 females) and 215 caregivers (56 males and 159 females) participated in the study and completed all assessments. The mean age of patients was 15.43 years (SD = 1.61); their mean age of onset was 14.05 years (SD = 1.92), and their mean length of education was 9.65 years (SD = 1.77). More than half of the patients were suffering from their first episode (*n* = 115, 53.5%) at the time of the assessment and most did not report a family history of psychiatric disorders (*n* = 203, 94.4%). For caregivers, the mean length of education was 12.32 years (SD = 3.40). Descriptive statistics of the HCL-33 and the HCL-33-EA are presented in Supplementary Table 1.

### Network Model of the 33-Item Hypomania Checklist

**FIGURE 1:**
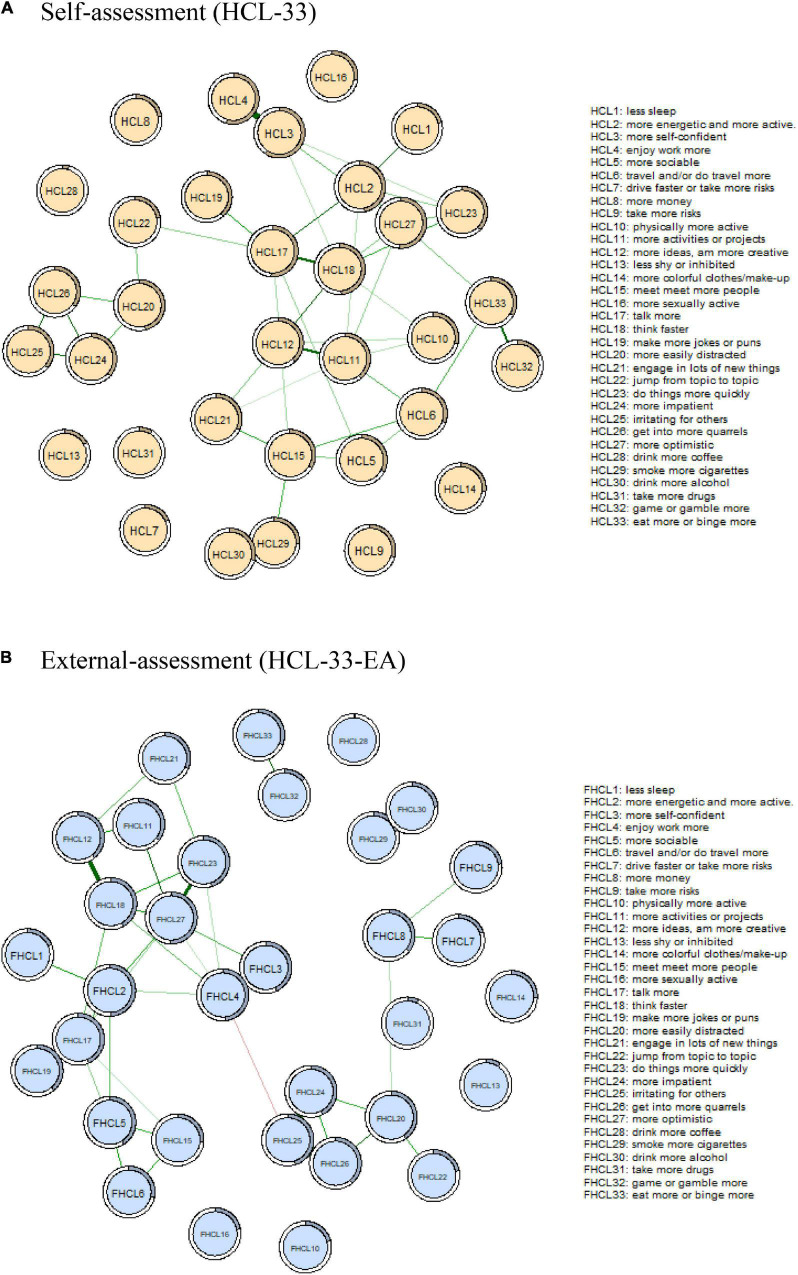
Comparison of network structure between HCL-33 **(A)** and HCL-33-EA **(B)**. In this diagram, nodes with stronger correlations are closer to each other. The thickness of an edge indicates the strength of the correlation. Green lines indicate positive associations. Red line indicates negative association. HCL-33, the 33-item Hypomania Checklist (self-assessment version); HCL-33-EA, the 33-item Hypomania Checklist (external assessment version).

Figure 1A shows the network structure of hypomanic symptoms measured by the HCL-33. The edge HCL29-HCL30 (“smoke more cigarettes” – “drink more alcohol,” edge weight = 2.172) showed the strongest positive connection in the model, followed by the edges HCL3-HCL4 (“more self-confident” – “enjoy work more,” edge weight = 1.848), and HCL11-HCL12 (“more activities” – “more ideas,” edge weight = 1.184).

**FIGURE 2:**
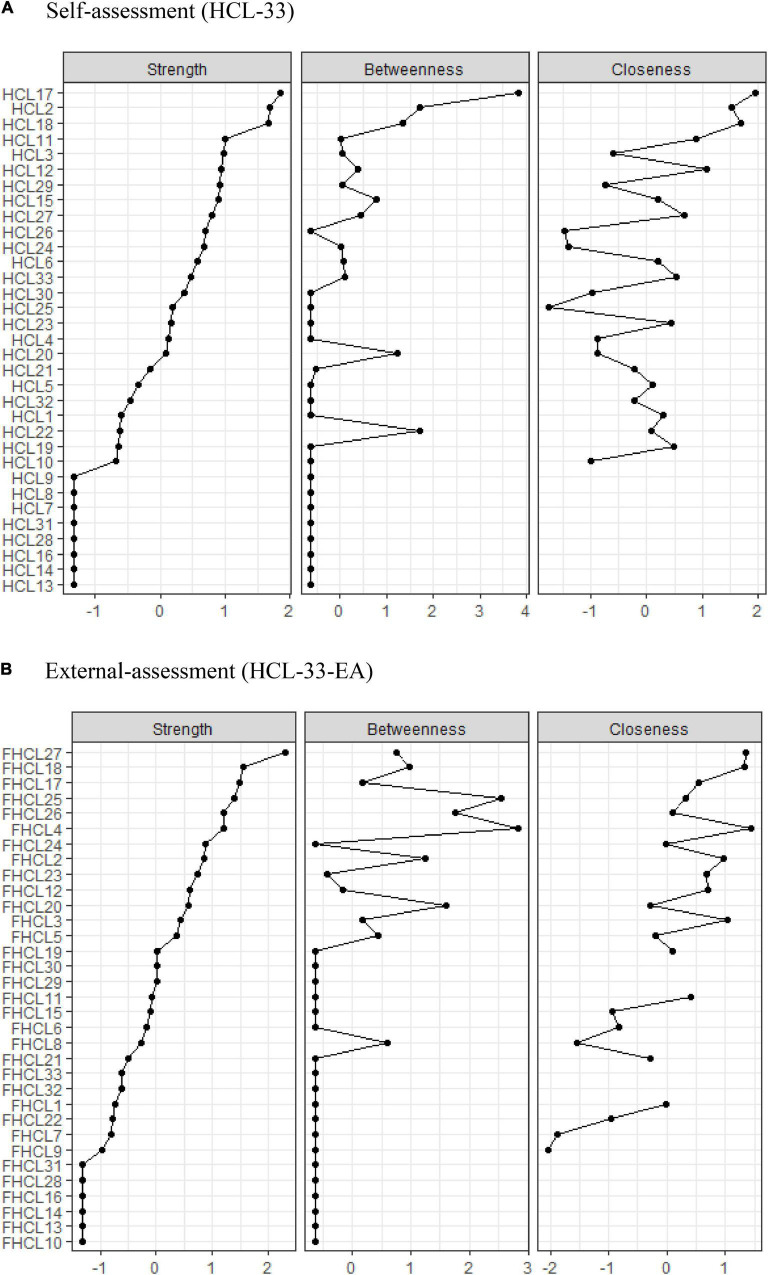
Comparison of the centrality indices between HCL-33 **(A)** and HCL-33-EA **(B)**. HCL-33, the 33-item Hypomania Checklist (self-assessment version); HCL-33-EA, the 33-item Hypomania Checklist (external assessment version).

The centrality plot indicated node HCL17 (“talk more,” node strength = 4.044) was the most central symptom in the HCL-33 symptom model, followed by nodes HCL2 (“more energetic,” node strength = 3.822), and HCL18 (“think faster,” node strength = 3.801). In contrast, nodes HCL7 (“drive faster”), HCL8 (“spend more money”), HCL9 (“take more risks”), HCL13 (“less shy”), HCL14 (“more colorful clothes/makeup”), HCL16 (“more sexually active”), HCL28 (“drink more coffee”), and HCL31 (“take more drugs”) were the least central responses in the symptom network (all node strength = 0). In addition, the predictability index showed that HCL3 (“more self-confident,” 59.1%), HCL4 (“enjoy work more,” 54.2%), and HCL17 (“talk more,” 51.0%) had the highest predictability in the network (Figure 2A and Table 1).

**TABLE 1:** Centrality of hypomania checklist items.

	HCL-33 (self-assessment version)				HCL-33-EA (external assessment version)			
	Strength	Betweenness	Closeness	Predictability	Strength	Betweenness	Closeness	Predictability
HCL1	0.934	0	0.008	0.218	0.627	0	0.005	0.188
HCL2	3.822	80	0.011	0.472	2.314	63	0.007	0.491
HCL3	2.927	23	0.007	0.591	1.884	27	0.007	0.432
HCL4	1.848	0	0.006	0.542	2.684	116	0.007	0.483
HCL5	1.270	0	0.008	0.338	1.800	36	0.005	0.406
HCL6	2.406	24	0.008	0.306	1.226	0	0.004	0.282
HCL7	0	0	NA	0.196	0.561	0	0.003	0.198
HCL8	0	0	NA	0.231	1.133	41	0.004	0.355
HCL9	0	0	NA	0.292	0.369	0	0.003	0.231
HCL10	0.835	0	0.006	0.291	0	0	NA	0.209
HCL11	2.947	22	0.010	0.381	1.316	0	0.006	0.359
HCL12	2.891	34	0.010	0.384	2.058	16	0.006	0.394
HCL13	0	0	NA	0.157	0	0	NA	0.091
HCL14	0	0	NA	0.232	0	0	NA	0.257
HCL15	2.829	48	0.008	0.337	1.302	0	0.004	0.265
HCL16	0	0	NA	0.273	0	0	NA	0.219
HCL17	4.044	152	0.012	0.510	2.998	27	0.006	0.434
HCL18	3.801	67	0.011	0.494	3.077	54	0.007	0.420
HCL19	0.864	0	0.009	0.390	1.430	0	0.006	0.386
HCL20	1.802	63	0.006	0.440	2.018	75	0.005	0.407
HCL21	1.481	3	0.008	0.324	0.866	0	0.005	0.313
HCL22	0.895	80	0.008	0.224	0.565	0	0.004	0.219
HCL23	1.915	0	0.009	0.378	2.185	7	0.006	0.447
HCL24	2.542	22	0.005	0.362	2.356	0	0.005	0.427
HCL25	1.939	0	0.005	0.444	2.881	106	0.006	0.447
HCL26	2.572	0	0.005	0.353	2.691	80	0.006	0.387
HCL27	2.709	36	0.009	0.509	3.867	46	0.007	0.507
HCL28	0	0	NA	0.029	0	0	NA	0.008
HCL29	2.847	23	0.007	0.253	1.425	0	NA	0.201
HCL30	2.172	0	0.006	0.289	1.425	0	NA	0.240
HCL31	0	0	NA	0.088	0	0	NA	0.067
HCL32	1.113	0	0.008	0.187	0.754	0	NA	0.143
HCL33	2.298	25	0.009	0.328	0.754	0	NA	0.310

*HCL-33, the 33-item Hypomania Checklist (self-assessment version); HCL-33-EA, the 33-item Hypomania Checklist (external assessment version).*

**FIGURE 3:**
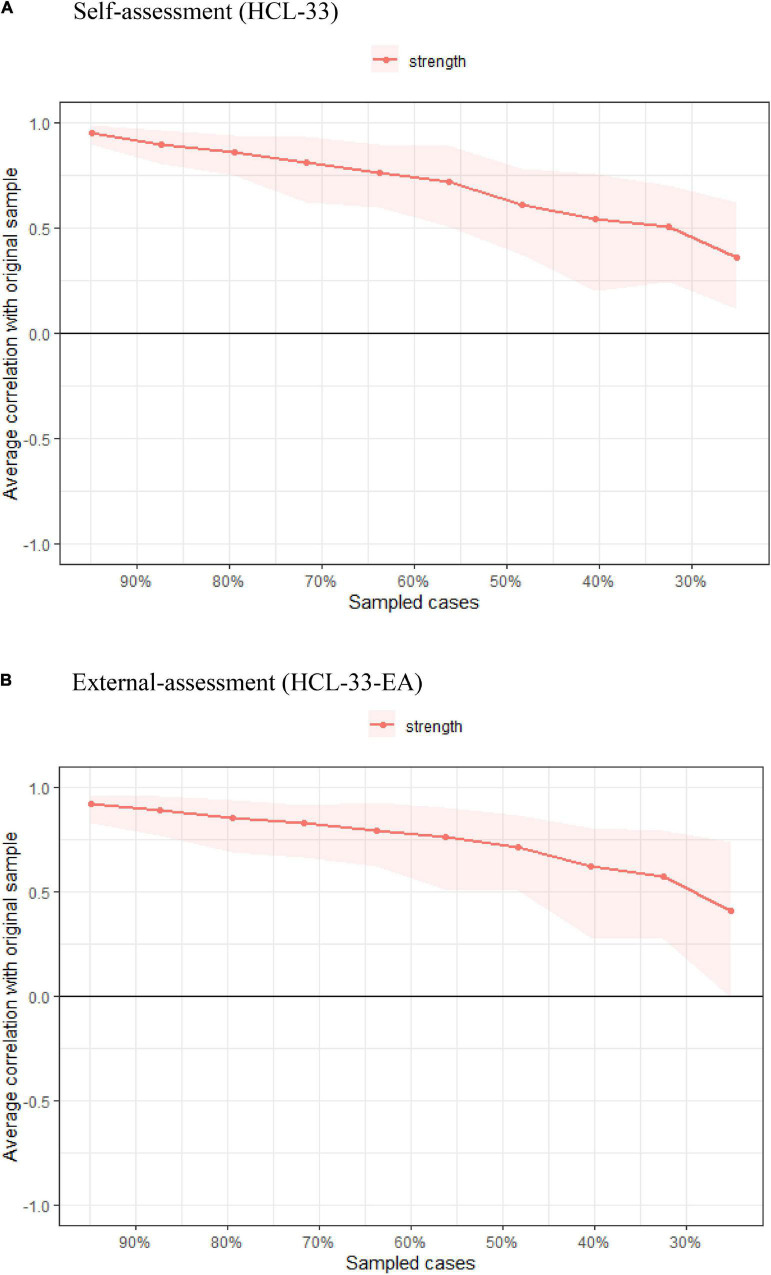
Comparison of stability of centrality indices between HCL-33 **(A)** and HCL-33-EA **(B)**. The *x*-axis represents the percentage of cases in the original sample used at each step. The *y*-axis represents the average of correlations between the centrality indices in the original network and the centrality indices in the networks that were re-estimated after dropping increasing percentages of cases. Color areas indicate 95% confidential intervals.

For stability of the HCL-33 network model, the case-dropping test showed that the CS coefficient for strength (0.284), exceeded the recommended threshold of 0.25, but was lower than 0.50 (Figure 3A). This indicated that the network model should be interpreted with caution as results might not be robust. Additionally, bootstrapped 95% CIs for estimated edge weights were relatively wide, suggesting comparatively low accuracy of edge strengths in the network (Supplementary Figure 1A). Plots of bootstrapped differences tests for HCL-33 edge weights and node strengths are presented in Supplementary Figures 2A, 3A.

### Network Model of the 33-Item Hypomania Checklist – External Assessment

Figure 1B shows the network structure of the HCL-33-EA. Similar to the HCL-33 model, the edge HCL3-HCL4 (“more self-confident” – “enjoy work more,” edge weight = 1.439) showed the strongest positive connection in the model, followed by edges HCL29-HCL30 (“smoke more cigarettes” – “drink more alcohol,” weight = 1.425), and HCL25-HCL26 (“irritating for others” – “get into more quarrels,” edge weight = 1.373). The only negative edge in the network was HCL4-HCL25 (“enjoy work more” – “irritating for others,” edge weight = −0.213).

The centrality plot showed that node HCL27 (“more optimistic,” node strength = 3.867) was the most central symptom in the HCL-33-EA network, followed by nodes HCL18 (“think faster,” node strength = 3.077), and HCL17 (“talk more,” node strength = 2.998). In contrast, nodes HCL10 (“physically more active”), HCL13 (“less shy”), HCL14 (“more colorful clothes/makeup”), HCL16 (“more sexually active”), HCL28 (“drink more coffee”), and HCL31 (“take more drugs”) were the least central symptoms in the network (all node strength = 0). In addition, the predictability index showed that HCL27 (“more optimistic,” 50.7%), HCL2 (“more energetic,” 49.1%), and HCL4 (“enjoy work more,” 48.3%) had the highest predictability in the network (Figure 2B and Table 1).

Similar to the HCL-33 model, the CS coefficient for strength in the HCL-33-EA network was also 0.284 (Figure 3B). Bootstrapped 95% CIs for estimated edge weights were relatively wide, suggesting low stability of the model and low accuracy of the edge weights (Supplementary Figure 1B). Plots of bootstrapped difference tests for HCL-33-EA edge weights and node strengths are presented in Supplementary Figures 2B, 3B.

### Network Comparison Between 33-Item Hypomania Checklist and 33-Item Hypomania Checklist – External Assessment Communities

The network comparison test showed that there were no significant differences in network structures of HCL-33 vs. HCL-33-EA symptom communities (*M* = 0.946, *P* = 0.931, Supplementary Figure 4A). Results of the global strength invariance test also indicated that the difference was not significant between the two network models generated from the HCL-33 and HCL-33-EA (HCL-33: 24.165 vs. HCL-33-EA: 18.991; S: 5.174, *P* = 0.274); as such, the total absolute connectivity among symptoms was similar for HCL-33 vs. HCL-33-EA communities (Supplementary Figure 4B). Tests of individual edge weights did not find significant differences between the two models (*P* all > 0.05, using Holm–Bonferroni corrections for multiple comparisons).

## Discussion

This is the first study to examine the structure of hypomanic symptoms using network analysis and the first to consider hypomania symptom communities, not only from the perspective of adolescent patients but also from the perspective of their caregivers (typically their parents). We found that “talk more” and “think faster” acted as central symptoms in network models of both the HCL-33 and HCL-33-EA but the most influential central symptom differed between these models (“talk more” in the HCL-33 model vs. “more optimistic” in the HCL-33-EA model). According to the Diagnostic and Statistical Manual of Mental Disorders, Fifth Edition (DSM-5) (33), unusual talkativeness, overall increases in energy, abnormally upbeat/inflated self-esteem, and racing thoughts are among the key characteristic behaviors of individuals with hypomania, in line with our findings. Conversely, “less shy,” “wear more colorful clothes/makeup,” “more sexually active,” “drink more coffee” and “take more drugs” were the least central nodes in networks of both the HCL-33 and HCL-33-EA. The reduced importance of these later symptoms and “driving faster” in networks of our sample was likely due to the reduced access adolescents have to alcohol/caffeine and drugs, sex with others, money for colorful clothes/makeup and a driver’s license/car compared to their adult counterparts.

Central symptoms may play critical roles in triggering the occurrence of a psychiatric disorder, maintaining the disorder, and predicting its course and clinical outcome (23). As such, central symptoms are potentially important as targets for treatment and prevention of psychiatric disorders (17, 23, 28). Our findings indicated that “talk more,” “more energetic,” “more optimistic,” and “think faster” were more influential than other hypomanic symptoms and had more connections with other symptoms in networks of both participants and caregivers. Therefore, it is possible that calculating a weighted total score of these central nodes or prioritizing these symptoms in clinical assessments (rather than total scores from entire hypomania symptom scales) may have utility in effectively capturing those hypomanic symptoms that are most crucial for understanding the severity of a BD (17, 23).

In this study, the edges “smoke more cigarettes” – “drink more alcohol,” and “more self-confident” – “enjoy work more” showed strong positive connections in network models of both the HCL-33 and HCL-33-EA. As such, these two edges were stable, strong, and tended to occur spontaneously. Significant links between alcohol use and smoking behaviors have been consistently reported in previous studies (34, 35). For example, heavy alcohol users smoke more frequently than do non-users, people tend to smoke more in settings where alcohol is served, and smokers are more likely than non-smokers to be binge drinkers (36). One reason for the strong smoking-alcohol use association is that alcohol and nicotine both increase dopaminergic activity levels in the human brain; therefore, co-administration of nicotine and alcohol may increase feelings of pleasure more than using either one of them alone (35). Furthermore, alcohol could enhance rewarding effects or calming effects of nicotine on frequent users (34). Finally, the current sample comprised Chinese adolescents often undergoing their initial episode of BPD. Hence, the link between smoking and alcohol use in this group may reflect low use levels of both substances compared to older BPD samples with chronic illness courses.

Regarding the link between self-confidence and work enjoyment, previous studies found that self-esteem was positively associated with job satisfaction (37–39). When an individual feels valued and fulfilled at work, he/she is more able to go above and beyond what is asked of him/her, which contributes to feelings of increased accomplishment and confidence (37, 38, 40).

The only negative edge found in the two networks was the connection between “irritating for others” and “work enjoyment” in the HCL-33-EA model; this association indicated that these two symptoms were not likely to occur simultaneously in patients from the perspective of their caregivers. Previous studies found that both positive and negative emotions are significantly associated with job satisfaction and performance (41). For example, anger emotions in the workplace could lead to aggressive and risky behaviors against colleagues, while sadness is related to elevations in job dissatisfaction. Job satisfaction has been defined as a positive emotional state resulting from an individual’s subjective experience with his/her job (42). When one enjoys his/her work, positive affect is more likely to be fostered, interpersonal relationships are less likely to be conflicted or irritating, and social support may help to increase enjoyment of one’s own work (43).

Previous research (44) has also found that when assessing health/disease status, external examiners (e.g., physicians, or caregivers) are more likely to focus on patients’ objective symptoms and diagnoses, whereas patients tend to focus more on their subjective symptoms, functional limitations, and quality of life. Therefore, there tends to be some discordance between patient self-assessments and external examiners’ assessments. Notably, the network comparison test in this study did not find significant differences at levels of network structure, global strength, or each specific edge between the patient HCL-33 and caregiver HCL-33-EA models. Hence, results provided preliminary evidence suggesting that HCL versions (self-report and external assessment) may not produce significantly different network model structures. Perhaps characteristics of adolescent-caregiver relationships (e.g., living together, a long-shared history, and often prolonged current contact) contributed to the lack of overall lack of disparity in observed network models but replications are needed to confirm the robustness of these assessment results.

A notable strength of the current study compared to a vast majority of network studies based exclusively on patient self-reports was its inclusion of a caregiver’s assessment of hypomania symptoms in each adolescent patient. Despite this strength and potential implications the research has for elucidating the structure of hypomania symptoms among adolescents diagnosed with BP, several limitations should also be noted. First, although network stabilities for both network models were acceptable, adolescent-caregiver sample sizes were relatively small as suggested by less-than-optimal CS-C results. Second, because data were drawn from a single China-based study-site, generalizability of findings to adolescent-caregiver dyads in other regions of China and other countries is unknown. Third, due to the cross-sectional study design, the evolution of hypomania symptom networks over time could not be determined from the present data. Fourth, BD subtypes were not diagnosed in this hospital; therefore, subtypes were not included for analyses in this study.

## Conclusion

In conclusion, the patient-assessed HCL-33 and caregiver-assessed HCL-33-EA generated similar hypomania symptom network structures and global strengths. The most and least influential hypomania symptoms in each of these network models were also somewhat similar: “talk more” and “think faster” emerged as important central symptoms in both HCL-33 and HCL-33-EA network models, though the single most influential symptom differed between the two models (i.e., “talk more” in the HCL-33, vs. “more optimistic” in the HCL-33-EA). Future studies with larger, more varied samples are warranted to confirm the accuracy and robustness of the hypomania symptom networks observed in this study.

## Data Availability Statement

The Medical Ethics Committee of the First Affiliated Hospital of Zhengzhou University that approved the study prohibits the authors from making the research dataset of clinical studies publicly available. Readers and all interested researchers may contact Y-TX (xyutly@gmail.com) for details. Y-TX will apply to the Medical Ethics Committee of the First Affiliated Hospital of Zhengzhou University for the release of the data.

## Ethics Statement

The studies involving human participants were reviewed and approved by the Medical Ethics Committee of the First Affiliated Hospital of Zhengzhou University. Written informed consent to participate in this study was provided by the participants’ legal guardian/next of kin.

## Author Contributions

SL and Y-TX: study design. YY, W-YZ, YZ, FH, TC, and DZ: data collection, analysis, and interpretation. YY and Y-TX: drafting of the manuscript. TJ: critical revision of the manuscript. All co-authors contributed to the approval of the final version for publication.

## Conflict of Interest

The authors declare that the research was conducted in the absence of any commercial or financial relationships that could be construed as a potential conflict of interest.

## Publisher’s Note

All claims expressed in this article are solely those of the authors and do not necessarily represent those of their affiliated organizations, or those of the publisher, the editors and the reviewers. Any product that may be evaluated in this article, or claim that may be made by its manufacturer, is not guaranteed or endorsed by the publisher.
