# Supplementary material for: Structure of Hypomanic Symptoms in Adolescents With Bipolar Disorders: A Network Approach
Source: Front Psychiatry. 2022 Apr 18;13:844699. doi: 10.3389/fpsyt.2022.844699 (PMC9058085; doi:10.3389/fpsyt.2022.844699)
Supplement: Supplementary file 1 [file Data_Sheet_1.pdf]

(A) Self-assessment (HCL-33)

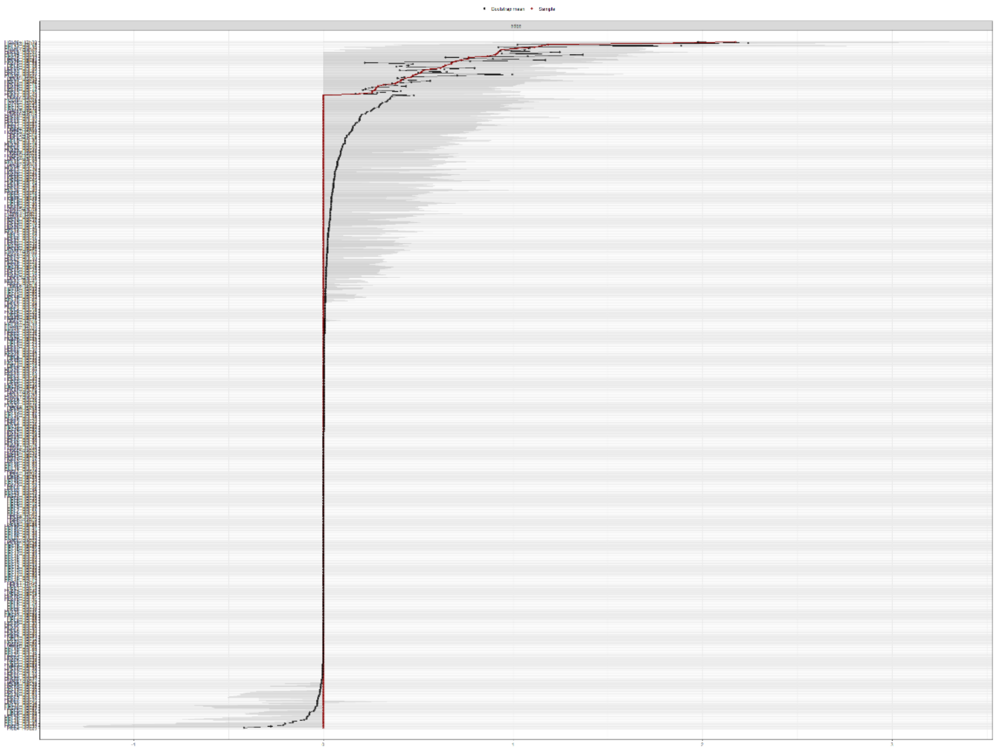

(B) External-assessment (HCL-33-EA)

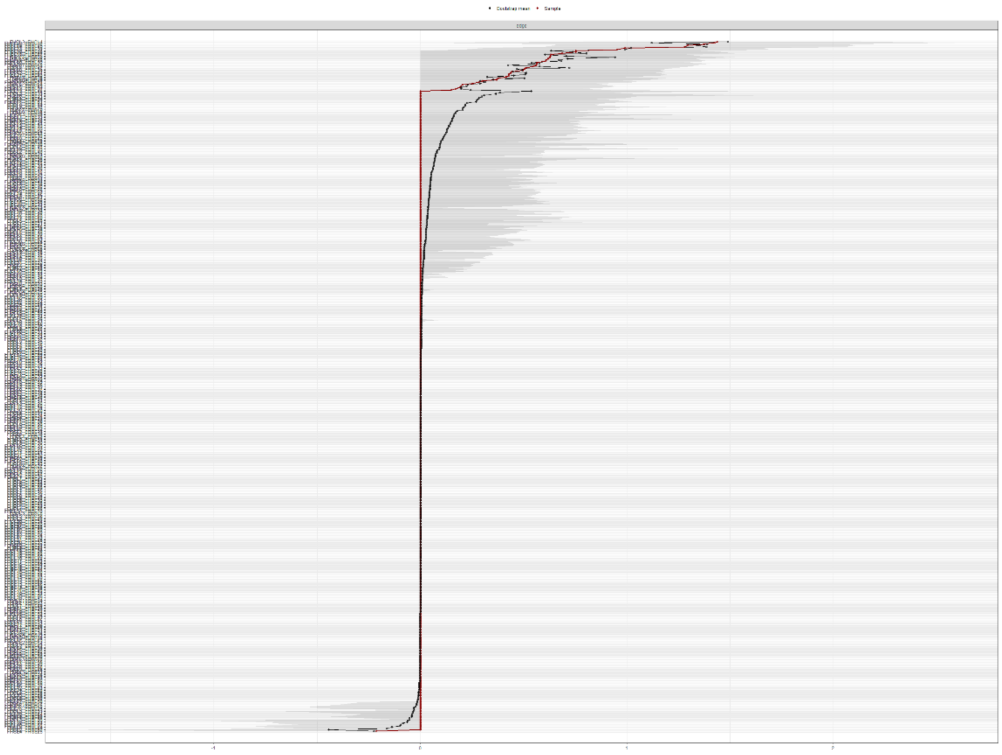

Supplementary Figure 1. Bootstrapped 95%CIs of estimated edge weights. Note: The red dots indicate the values of each edge weight, ordered from the highest to the lowest edge-weight values. The gray area represents the 95% CIs of edge weights, estimated with the non-parametric bootstrap procedure. Wide intervals indicate lower stability and narrow intervals indicate higher stability.

(A) Self-assessment (HCL-33)

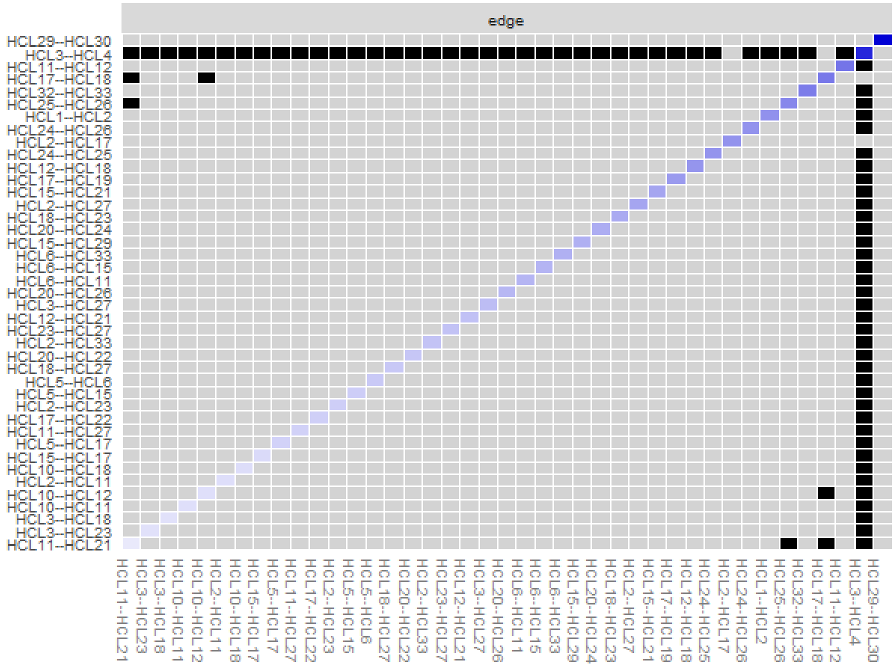

(B) External-assessment (HCL-33-EA)

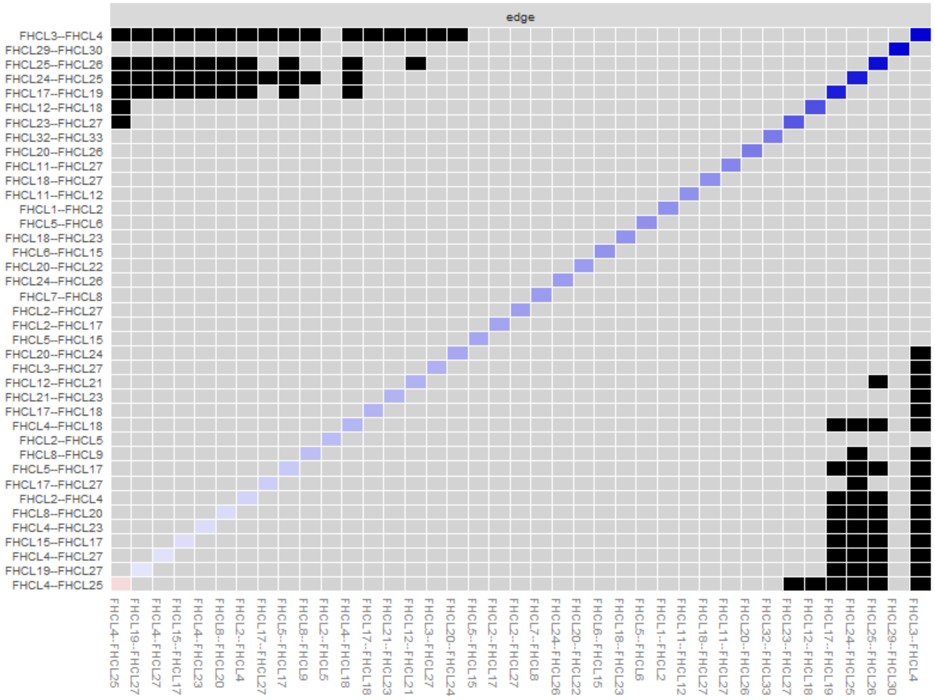

Supplementary Figure 2. Estimation of edge weight difference by bootstrapped difference test. Note: Gray boxes represent the edges do not significantly differ from one-another, and black boxes represent edges that do differ significantly from one-another. The diagonal line indicates the strength of edge-weights, shifting from red (representing negative associations) to dark blue (representing positive associations).

(A) Self-assessment (HCL-33)

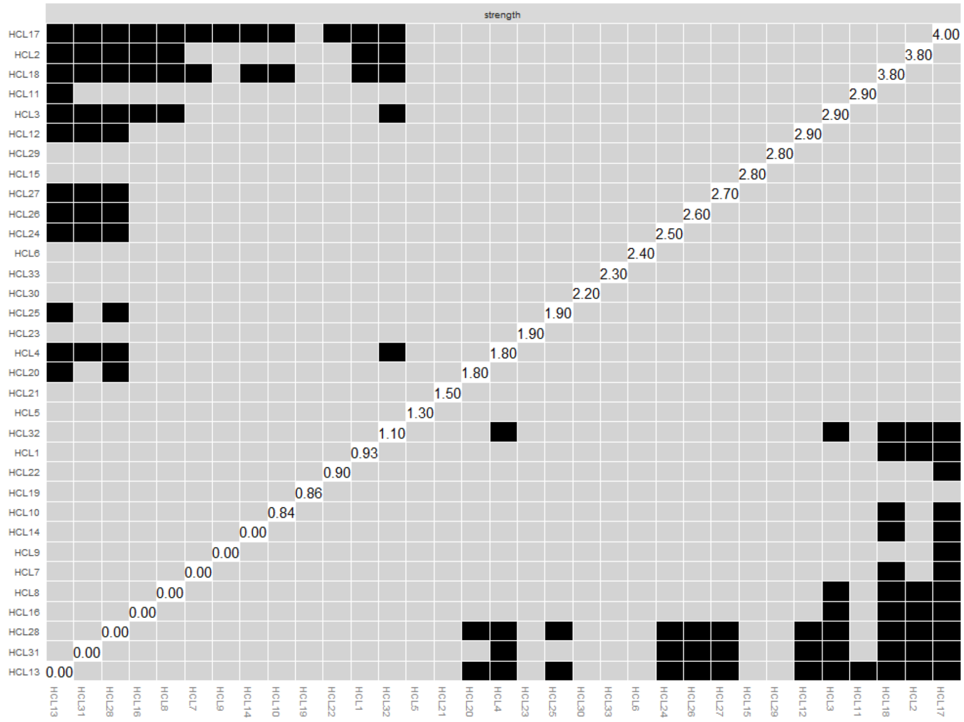

(B) External-assessment (HCL-33-EA)

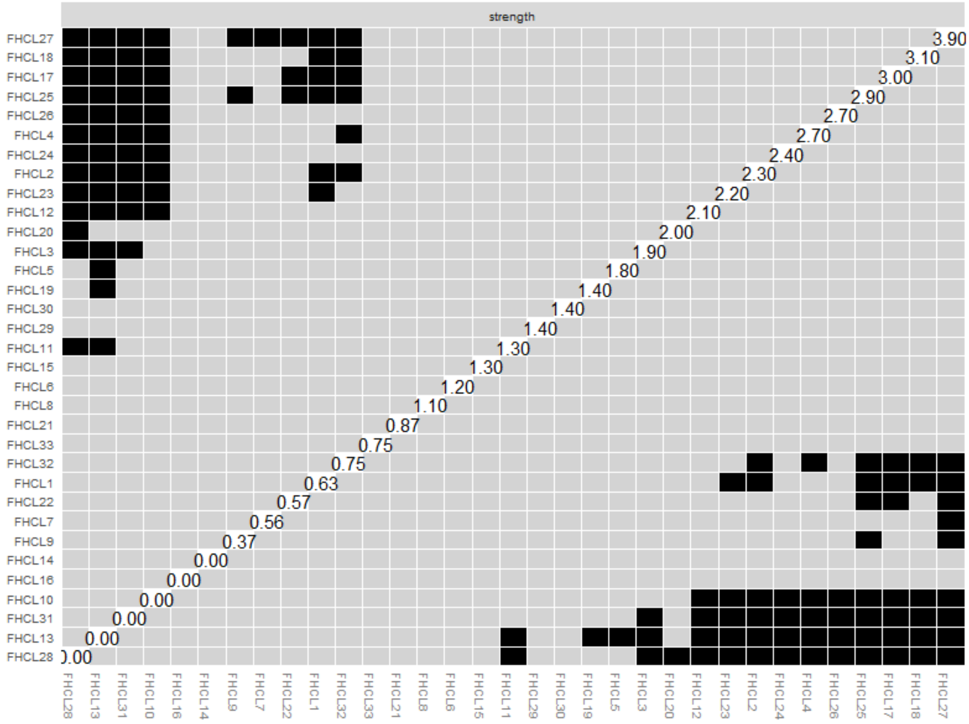

Supplementary Figure 3. Nonparametric bootstrapped difference test for strength. Note: Gray boxes indicate no difference between nodes, whereas black boxes indicate significant difference (α = 0.05). Values reported in the diagonal represent the strength values of each node.

(A) Network structure invariance test

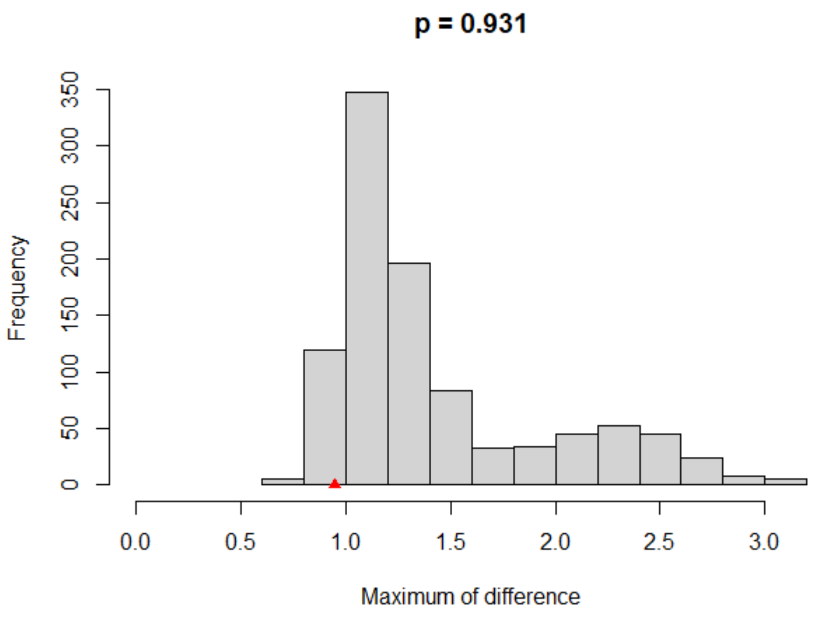

(B) Global strength invariance test

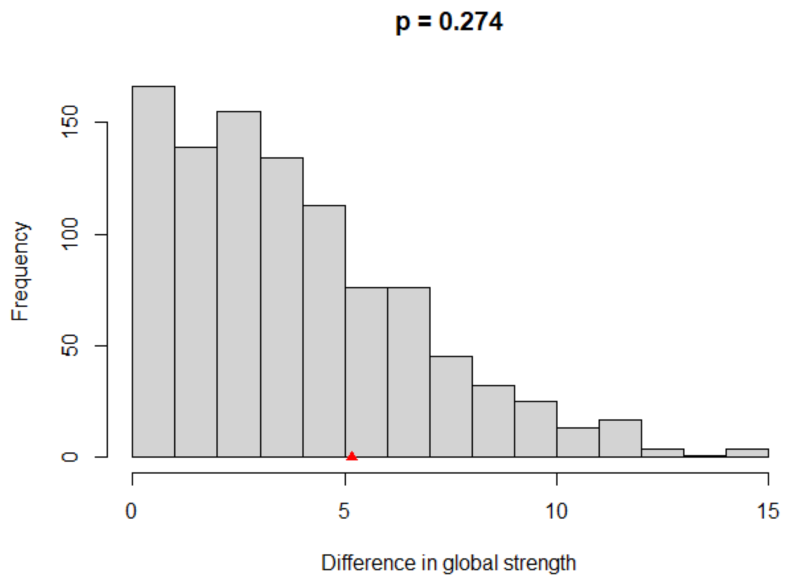

Supplementary Figure 4. Comparison of network properties between HCL-33 and HCL-33-EA. Note: (A): a plot of bootstrap value of the maximum difference in any of the edge weights (1000 permutations); (B): a plot of bootstrap value of the difference in network global strength (1000 permutations). HCL-33: the 33-item Hypomania Checklist (self-assessment version); HCL-33-EA: the 33-item Hypomania Checklist (external assessment version).

Supplementary Table 1. Descriptive statistics of HCL-33 and HCL-33-EA

	HCL-33					HCL-33-EA				
	**N**	**Minimum**	**Maximum**	**Mean**	**Std. Deviation**	**N**	**Minimum**	**Maximum**	**Mean**	**Std. Deviation**
**HCL1**	215	0.00	1.00	0.57	0.50	215	0.00	1.00	0.48	0.50
**HCL2**	215	0.00	1.00	0.69	0.46	215	0.00	1.00	0.61	0.49
**HCL3**	215	0.00	1.00	0.49	0.50	215	0.00	1.00	0.47	0.50
**HCL4**	215	0.00	1.00	0.51	0.50	215	0.00	1.00	0.47	0.50
**HCL5**	215	0.00	1.00	0.58	0.49	215	0.00	1.00	0.51	0.50
**HCL6**	215	0.00	1.00	0.50	0.50	215	0.00	1.00	0.44	0.50
**HCL7**	215	0.00	1.00	0.32	0.47	215	0.00	1.00	0.21	0.41
**HCL8**	215	0.00	1.00	0.60	0.49	215	0.00	1.00	0.53	0.50
**HCL9**	215	0.00	1.00	0.42	0.49	215	0.00	1.00	0.31	0.46
**HCL10**	215	0.00	1.00	0.42	0.49	215	0.00	1.00	0.34	0.48
**HCL11**	215	0.00	1.00	0.57	0.50	215	0.00	1.00	0.52	0.50
**HCL12**	215	0.00	1.00	0.56	0.50	215	0.00	1.00	0.53	0.50
**HCL13**	215	0.00	1.00	0.51	0.50	215	0.00	1.00	0.43	0.50
**HCL14**	215	0.00	1.00	0.33	0.47	215	0.00	1.00	0.29	0.45
**HCL15**	215	0.00	1.00	0.44	0.50	215	0.00	1.00	0.38	0.49
**HCL16**	215	0.00	1.00	0.22	0.41	215	0.00	1.00	0.13	0.34
**HCL17**	215	0.00	1.00	0.71	0.45	215	0.00	1.00	0.65	0.48
**HCL18**	215	0.00	1.00	0.57	0.50	215	0.00	1.00	0.53	0.50
**HCL19**	215	0.00	1.00	0.64	0.48	215	0.00	1.00	0.58	0.50
**HCL20**	215	0.00	1.00	0.56	0.50	215	0.00	1.00	0.43	0.50
**HCL21**	215	0.00	1.00	0.54	0.50	215	0.00	1.00	0.47	0.50
**HCL22**	215	0.00	1.00	0.68	0.47	215	0.00	1.00	0.55	0.50
**HCL23**	215	0.00	1.00	0.51	0.50	215	0.00	1.00	0.44	0.50
**HCL24**	215	0.00	1.00	0.66	0.47	215	0.00	1.00	0.63	0.48
**HCL25**	215	0.00	1.00	0.49	0.50	215	0.00	1.00	0.49	0.50
**HCL26**	215	0.00	1.00	0.44	0.50	215	0.00	1.00	0.39	0.49
**HCL27**	215	0.00	1.00	0.53	0.50	215	0.00	1.00	0.47	0.50
**HCL28**	215	0.00	1.00	0.13	0.33	215	0.00	1.00	0.11	0.31
**HCL29**	215	0.00	1.00	0.08	0.27	215	0.00	1.00	0.07	0.26
**HCL30**	215	0.00	1.00	0.14	0.35	215	0.00	1.00	0.11	0.32
**HCL31**	215	0.00	1.00	0.12	0.33	215	0.00	1.00	0.12	0.32

**HCL32**	215	0.00	1.00	0.27	0.44	215	0.00	1.00	0.25	0.43
**HCL33**	215	0.00	1.00	0.40	0.49	215	0.00	1.00	0.37	0.48
**Total Score**	215	0.00	29.00	15.19	6.11	215	0.00	29.00	13.29	5.99

Note: HCL-33: the 33-item Hypomania Checklist (self-assessment version); HCL-33-EA: the 33-item Hypomania Checklist (external assessment version).
